# Supplementary material for: Towards Unraveling the Histone Code by Fragment Blind Docking
Source: Int J Mol Sci. 2019 Jan 19;20(2):422. doi: 10.3390/ijms20020422 (PMC6358888; doi:10.3390/ijms20020422)
Supplement: Supplementary file 1 [file ijms-20-00422-s001.zip › ijms-420522 suppl revised/supplementary_tables.pdf]

Supplementary Tables

Towards unraveling the histone code by fragment blind docking

Mónika Bálint[1], István Horváth[2], Nikolett Mészáros[3], Csaba Hetényi[1,*]

[1]Department of Pharmacology and Pharmacotherapy, Medical School, University of Pécs, Szigeti út 12, 7624 Pécs, Hungary.
[2]Chemistry Doctoral School, University of Szeged, Dugonics tér 13, 6720 Szeged, Hungary.
[3]Department of Biochemistry, Eötvös Loránd University, Pázmány Péter sétány 1/C, 1117 Budapest, Hungary.

*Corresponding author.

Supplementary Table 1 Docking results obtained for each peptide fragment

PDB	Fragment type	Fragment sequence	# Wrapping cycle	# Rank	Best RMSD
2KE1	N2	AR-NHMe	1	4	1.9
2KE1	N2	Ac-TK-NHMe	1	1	2.8
2KE1	N2	Ac-QT-NHMe	5	5	6.7
2KE1	N2	Ac-AR-NHMe	1	4	6.4
2KE1	N2	Ac-KS	5	23	6.3
2KE1	N3	ART-NHMe	1	5	5.4
2KE1	N3	Ac-KQT-NHMe	1	1	2.4
2KE1	N3	Ac-AR-NHMe	1	4	6.4
2KE1	N3	Ac-KS	5	23	6.3
2KE1	N4	ARTK-NHMe	1	6	9.6
2KE1	N4	Ac-QTAR-NHMe	5	1	8.1
2KE1	N4	Ac-KS	5	23	6.3
2KE1	C2	AR-NHMe	1	4	1.9
2KE1	C2	Ac-TK-NHMe	1	1	2.8
2KE1	C2	Ac-QT-NHMe	5	5	6.7
2KE1	C2	Ac-AR-NHMe	1	4	6.4
2KE1	C2	Ac-KS	5	23	6.3
2KE1	C3	AR-NHMe	1	4	1.9
2KE1	C3	Ac-TK-NHMe	1	1	2.8
2KE1	C3	Ac-QTA-NHMe	2	2	5.2
2KE1	C3	Ac-RKS	1	7	6.2
2KE1	C4	AR-NHMe	1	4	1.9
2KE1	C4	Ac-TKQT-NHMe	1	1	7.8
2KE1	C4	Ac-ARKS	1	2	9.1
2PVC	N2	AR-NHMe	2	5	3.1
2PVC	N2	Ac-TK-NHME	1	3	2.8
2PVC	N2	Ac-QTA	2	17	4.5
2PVC	N3	ART-NHMe	2	2	6.7
2PVC	N3	Ac-KQTA	1	1	3.0
2PVC	N4	ARTK-NHMe	2	7	7.3
2PVC	N4	Ac-QTA	2	17	4.5
2PVC	C2	ART-NHMe	2	2	6.7
2PVC	C2	Ac-KQ-NHMe	1	2	1.7
2PVC	C2	Ac-TA	6	4	6.8
2PVC	C3	ARTK-NHMe	2	7	7.3
2PVC	C3	Ac-QTA	2	17	4.5
2PVC	C4	ART-NHMe	2	2	6.7
2PVC	C4	Ac-KQTA	1	1	3.0
2PVC	N2	AR-NHMe	2	5	3.1
2PVC	N2	Ac-TK-NHMe	1	3	2.8
2PVC	N2	Ac-QTA	2	17	4.5
2PVC	N3	ART-NHMe	2	2	6.7
2PVC	N3	Ac-KQTA	1	1	3.0
2PVC	N4	ARTK-NHMe	2	7	7.3

PDB	Fragment type	Fragment sequence	# Wrapping cycle	# Rank	Best RMSD
2PVC	N4	Ac-QTA	2	17	4.5
2PVC	C2	ART-NHMe	2	2	6.7
2PVC	C2	Ac-KQ-NHMe	1	2	1.7
2PVC	C2	Ac-TA	6	4	6.8
2PVC	C3	ARTK-NHMe	2	7	7.3
2PVC	C3	Ac-QTA	2	17	4.5
2PVC	C4	ART-NHMe	2	2	6.7
2PVC	C4	Ac-KQTA	1	1	3.0
3QLA	N2	AR-NHMe	1	1	7.0
3QLA	N2	Ac-TK-NHMe	5	5	7.2
3QLA	N2	Ac-QT-NHMe	1	12	2.0
3QLA	N2	Ac-AR-NHMe	7	1	7.2
3QLA	N2	Ac-K(Me_3)S	1	2	3.7
3QLA	N3	ART-NHMe	1	6	5.4
3QLA	N3	Ac-KQT-NHMe	1	3	6.7
3QLA	N3	Ac-ARK(Me_3)S	1	6	9.2
3QLA	N4	ARTK-NHMe	1	1	3.2
3QLA	N4	Ac-QTAR-NHMe	3	1	8.9
3QLA	N2	AR-NHMe	1	1	7.0
3QLA	N2	Ac-TK-NHMe	5	5	7.2
3QLA	N2	Ac-QT-NHMe	1	12	2.0
3QLA	N2	Ac-AR-NHMe	7	1	7.2
3QLA	C3	Ac-QTA-NHMe	1	17	6.4
3QLA	C3	Ac-RK(Me_3)S	1	3	6.8
3QLA	C4	Ac-TKQT-NHMe	2	3	8.0
4LK9	N2	AR-NHMe	1	1	2.9
4LK9	N2	Ac-TK-NHMe	1	4	4.0
4LK9	N2	Ac-QT-NHMe	6	3	6.2
4LK9	N2	Ac-AR-NHMe	7	1	5.7
4LK9	N2	Ac-KS-NHMe	4	2	6.5
4LK9	N2	Ac-TGG	6	4	7.0
4LK9	N3	ART-NHMe	1	1	6.1
4LK9	N3	Ac-KQT-NHMe	1	12	6.2
4LK9	N3	Ac-ARK-NHMe	4	1	5.3
4LK9	N3	Ac-ST-NHMe	6	4	4.8
4LK9	N3	Ac-GG	8	6	6.4
4LK9	N4	ARTK-NHMe	1	1	7.0
4LK9	N4	Ac-QTAR-NHMe	1	1	9.0
4LK9	N4	Ac-KST-NHMe	3	3	4.8
4LK9	N4	Ac-GG	8	6	6.4
4LK9	C2	ART-NHMe	1	1	6.1
4LK9	C2	Ac-KQ-NHMe	1	3	5.3
4LK9	C2	Ac-TA-NHMe	8	1	5.8
4LK9	C2	Ac-RK-NHMe	2	6	7.8
4LK9	C2	Ac-ST-NHMe	6	4	4.8

PDB	Fragment type	Fragment sequence	# Wrapping cycle	# Rank	Best RMSD
4LK9	C2	Ac-GG	8	6	6.4
4LK9	C3	AR-NHMe	1	1	2.9
4LK9	C3	Ac-TK-NHMe	1	4	4.1
4LK9	C3	Ac-QTA-NHMe	1	8	8.5
4LK9	C3	Ac-RKS-NHMe	1	1	10.5
4LK9	C3	Ac-TGG	6	4	7.0
4LK9	C4	AR-NHMe	1	1	2.9
4LK9	C4	Ac-TKQ-NHMe	1	5	4.3
4LK9	C4	Ac-TARK-NHMe	1	1	9.6
4LK9	C4	Ac-STGG	6	8	7.1
5TDW	N2	AR-NHMe	1	1	1.7
5TDW	N2	Ac -TK(Me_3)-NHMe	2	8	7.7
5TDW	N2	Ac-QT-NHMe	5	3	7.5
5TDW	N2	Ac-AR-NHMe	1	5	11.8
5TDW	N2	Ac-KST	8	3	7.2
5TDW	N3	Ac-NHMe	1	1	5.1
5TDW	N3	Ac-K(Me_3)QT-NHMe	2	4	9.8
5TDW	N3	Ac-ARK-NHMe	1	2	12.7
5TDW	N3	Ac-ST	17	42	5.5
5TDW	N4	ARTK(Me_3)-NHMe	1	1	7.7
5TDW	N4	Ac-QTAR-NHMe	5	2	11.1
5TDW	C2	Ac-K(Me_3)Q-NHMe	1	1	8.2
5TDW	C2	Ac-TA-NHMe	7	4	5.7
5TDW	C2	Ac-RK-NHMe	1	6	13.6
5TDW	C3	Ac-TK(Me_3)Q-NHMe	2	8	8.3
5TDW	C3	Ac-TAR-NHMe	2	2	8.7
5TDW	C4	Ac-K(Me_3)QTA-NHMe	2	2	8.9
5TDW	C4	Ac-RKST	10	19	8.0

Supplementary Table 2 Interacting residues of target proteins

H3 amino acid	2ke1	2pvc	3qla	4lk9	5tdw
A1	P331<br>G333	M137<br>I107	D233<br>-	G303<br>-	P40<br>D41
R2	C310<br>N295<br>C311<br>D312<br>-	L105<br>I107<br>-<br>-<br>-	C231<br>-<br>-<br>-<br>-	L279<br>F280<br>D282<br>D285<br>C281	I17<br>Q18<br>N23<br>C19<br>-
T3	L308<br>I330	L105<br>-	L229	M278<br>D276	T16<br>I36
K4	L308<br>G306<br>N295<br>E296<br>D297	L105<br>D90<br>D88<br>Q93<br>-	L229<br>G227<br>D212<br>D217<br>-	A275<br>M278<br>I260<br>E261<br>K263	T16<br>F15<br>-<br>-<br>-
Q5	G306<br>-	E103<br>-	G227<br>N228	A275<br>D276	G14<br>-
T6	G306<br>D297<br>L308<br>-	-<br>-<br>-<br>-	G227<br>E218<br>G226<br>G227	-<br>-<br>-<br>-	G14<br>-<br>-<br>-
A7	G305	-	G226	-	-
R8	E307<br>D307	-<br>-	E225<br>-	-<br>-	-<br>-
K9	E298<br>-<br>-<br>-	-<br>-<br>-<br>-	E225<br>Y203<br>A224<br>Q219	-<br>-<br>-<br>-	-<br>-<br>-<br>-
S10	D304	-	-	L243	-
T11	-<br>-	-<br>-	-<br>-	F211<br>I260	-<br>-

Supplementary Table 3 Values of E_{inter} and N_{inter}

H3 amino acid	2ke1		2pvc		3qla		4lk9		5tdw	
	E_{inter}	N_{inter}	E_{inter}	N_{inter}	E_{inter}	N_{inter}	E_{inter}	N_{inter}	E_{inter}	N_{inter}
A1	-10.4	2	-10.8	2	-20.2	1	-11.0	1	-15.7	2
R2	-16.0	4	-7.2	2	-20.5	1	-28.4	5	-21.5	4
T3	-11.4	2	-5.9	1	-6.8	1	-15.3	2	-8.2	2
K4	-15.5	5	-26.8	4	-23.2	4	-20.5	5	-17.4	2
Q5	-8.3	1	-6.2	1	-5.8	2	-9.0	2	-8.8	1
T6	-10.1	3	-5.3		-10.4	4	-2.2	0	-3.0	1
A7	-4.4	1	-0.9		-2.6	1	-4.2	0	-0.2	0
R8	-8.8	2			-8.7	1	-15.4	0	-0.9	0
K9	-4.4	1			-22.0	4	0.9	0	-0.4	0
S10	-2.9	1			-0.2	0	-4.2	1	-0.0	0
T11							-8.1	2	0.2	0
G12							-1.0	0		
G13							-3.4	0		

Supplementary Table 4 Command line parameters of FragmentMerge*

Parameter	Description
-i	Input directory
-p	Output and report path
-m	Minimum distance between all heavy atoms
-x	Maximum distance between all heavy atoms
-n	Minimum distance between the N and C atoms to connect
-a	Angle of the rotation

*Command line:

java FragmentMerge –p <string> –m <real> –x <real> –n <real> –a <real> –i <string>

Contents of sample input text file with the name of the system and the fragments:

C2
AR
TK
QT
AR
KS

Supplementary Table 5 Example report file produced by FragmentMerge

System
2KE1

ID
C2

Remark
\home\user\CBDD\PDB\LINKED\2KE1\C2\LINKING\

Ligand
ARTKQTARKS

Parameters
dCN,max = 6.0
dCN,min = 0.75
alpha = 1.0
dall = 0.75

Path of input pools (fragment copies)
\home\user\CBDD\PDB\LINKED\2KE1\C2\LINKING\INPUT

List of fragments; count of copies
AR1 28
TK2 39
QT3 69
AR4 31
KS5 81

Path of output pools (linked structures)
\home\user\CBDD\PDB\LINKED\2KE1\C2\LINKING\OUTPUT\

List of output pools; count of linked peptides

Pentads 2
\home\user\CBDD\PDB\LINKED\2KE1\C2\LINKING\OUTPUT\Pentads\
AR25 - TK04 - QT07 - AR06 - KS14
AR17 - TK04 - QT07 - AR06 - KS14

Tetrads 2
\home\user\CBDD\PDB\LINKED\2KE1\C2\LINKING\OUTPUT\Tetrads\ 2018.10.13.14.44.16\part2-5
TK02 - QT51 - AR02 - KS27
TK04 - QT07 - AR06 - KS14

Triads 20
\home\user\CBDD\PDB\LINKED\2KE1\C2\LINKING\OUTPUT\Triads\
AR22 - TK28 - QT56
AR22 - TK28 - QT18
AR14 - TK38 - QT68
TK17 - QT52 - AR08
TK21 - QT35 - AR14
TK02 - QT51 - AR21
TK07 - QT14 - AR03
QT57 - AR25 - KS42
QT07 - AR06 - KS14
QT50 - AR26 - KS30
QT58 - AR27 - KS52
QT58 - AR27 - KS18
QT03 - AR10 - KS38
QT03 - AR10 - KS56
QT39 - AR15 - KS51
QT39 - AR15 - KS47
QT51 - AR02 - KS27
QT32 - AR15 - KS51
QT32 - AR15 - KS47
QT32 - AR28 - KS25

Dyads (Pairs) 59
\home\user\CBDD\PDB\LINKED\2KE1\C2\LINKING\OUTPUT\Dyads\
AR17 - TK24
AR18 - TK39
AR04 - TK01
AR20 - TK24
AR07 - TK39
AR27 - TK24
TK22 - QT41
TK05 - QT67
TK36 - QT17
TK35 - QT55
TK29 - QT36
TK17 - QT16
TK06 - QT30
TK37 - QT38
TK09 - QT49
TK20 - QT60
TK19 - QT27
TK22 - QT25
TK38 - QT68
TK23 - QT30
TK10 - QT25
TK13 - QT37
TK34 - QT19
TK10 - QT41
TK28 - QT56
TK05 - QT20
TK28 - QT18
QT33 - AR30
QT51 - AR21
QT09 - AR17
QT66 - AR23
QT53 - AR19
QT29 - AR18
QT59 - AR04

QT42 - AR18
QT52 - AR08
QT62 - AR05
QT35 - AR14
QT58 - AR23
QT08 - AR16
QT31 - AR08
QT14 - AR03
AR06 - KS14
AR26 - KS30
AR28 - KS25
AR27 - KS52
AR13 - KS01
AR02 - KS27
AR15 - KS51
AR10 - KS38
AR12 - KS14
AR27 - KS18
AR10 - KS56
AR01 - KS31
AR25 - KS42
AR12 - KS10
AR24 - KS38
AR11 - KS28
AR15 - KS47
