# Supplementary figures and images for: Towards Unraveling the Histone Code by Fragment Blind Docking
Source: Int J Mol Sci. 2019 Jan 19;20(2):422. doi: 10.3390/ijms20020422 (PMC6358888; doi:10.3390/ijms20020422)

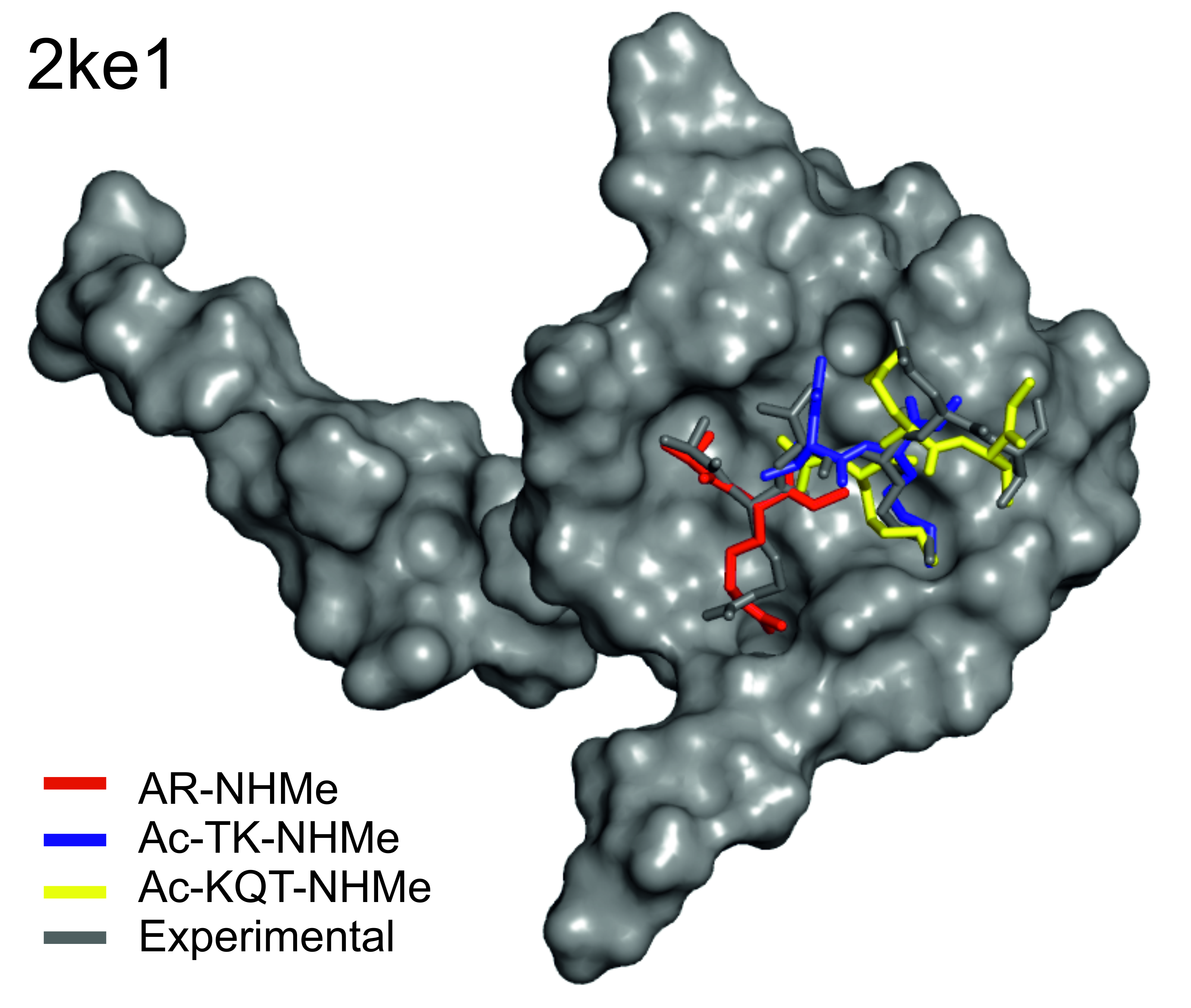

Supplement: Supplementary file 1 [file ijms-20-00422-s001.zip › ijms-420522 suppl revised/supplementary_figs/supplementary_fig_1.tif]

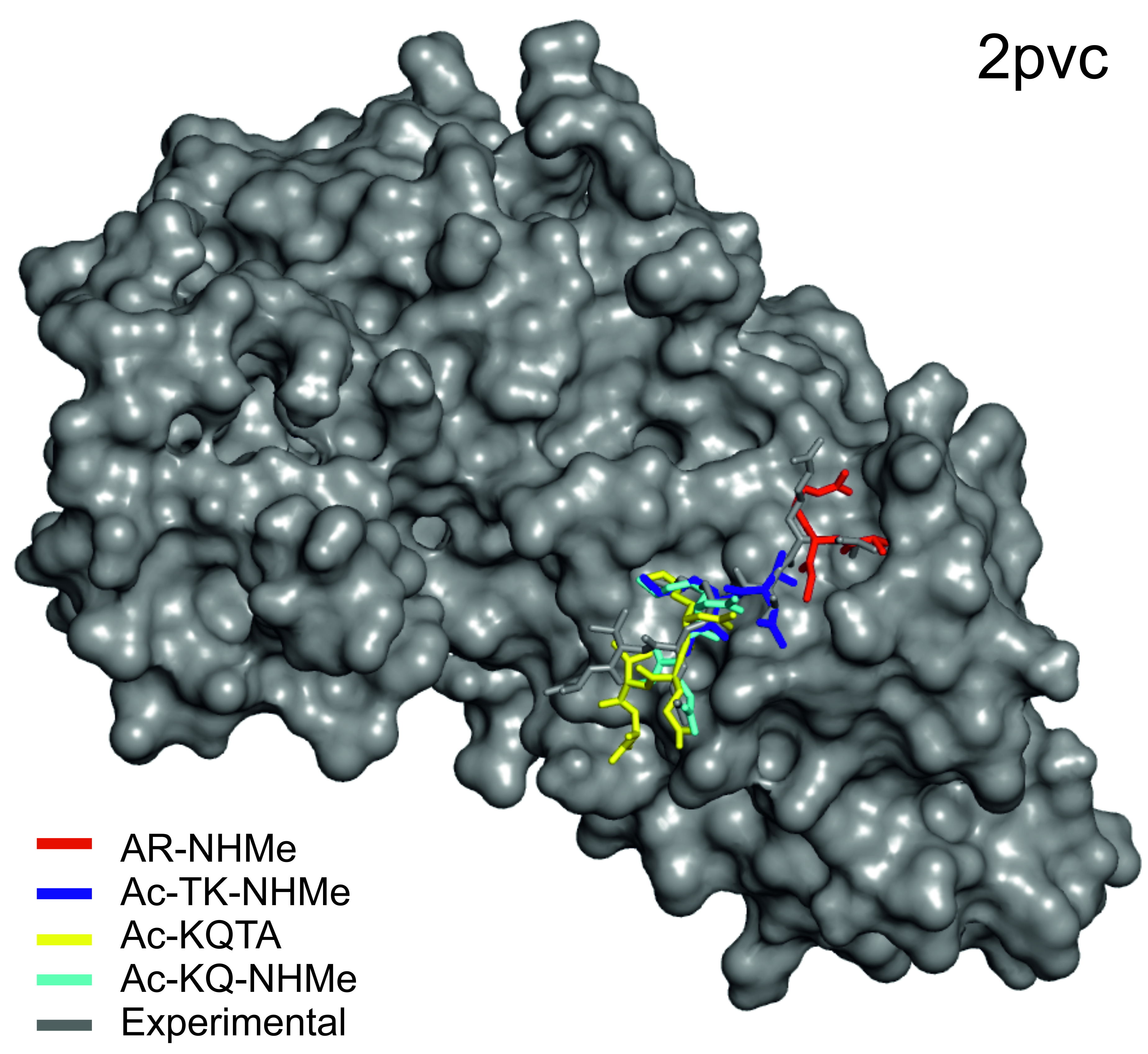

Supplement: Supplementary file 1 [file ijms-20-00422-s001.zip › ijms-420522 suppl revised/supplementary_figs/supplementary_fig_2.tif]

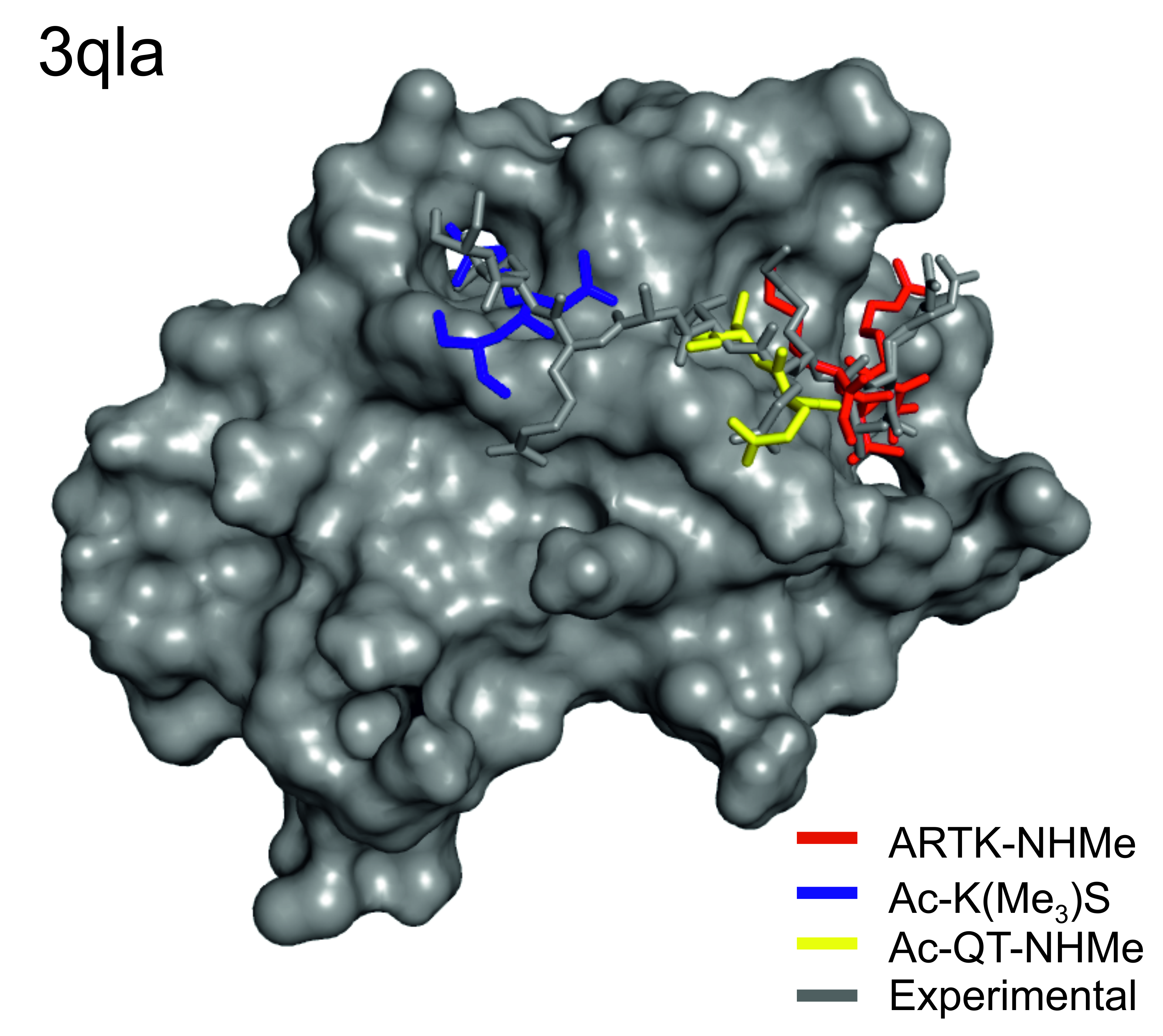

Supplement: Supplementary file 1 [file ijms-20-00422-s001.zip › ijms-420522 suppl revised/supplementary_figs/supplementary_fig_3.tif]

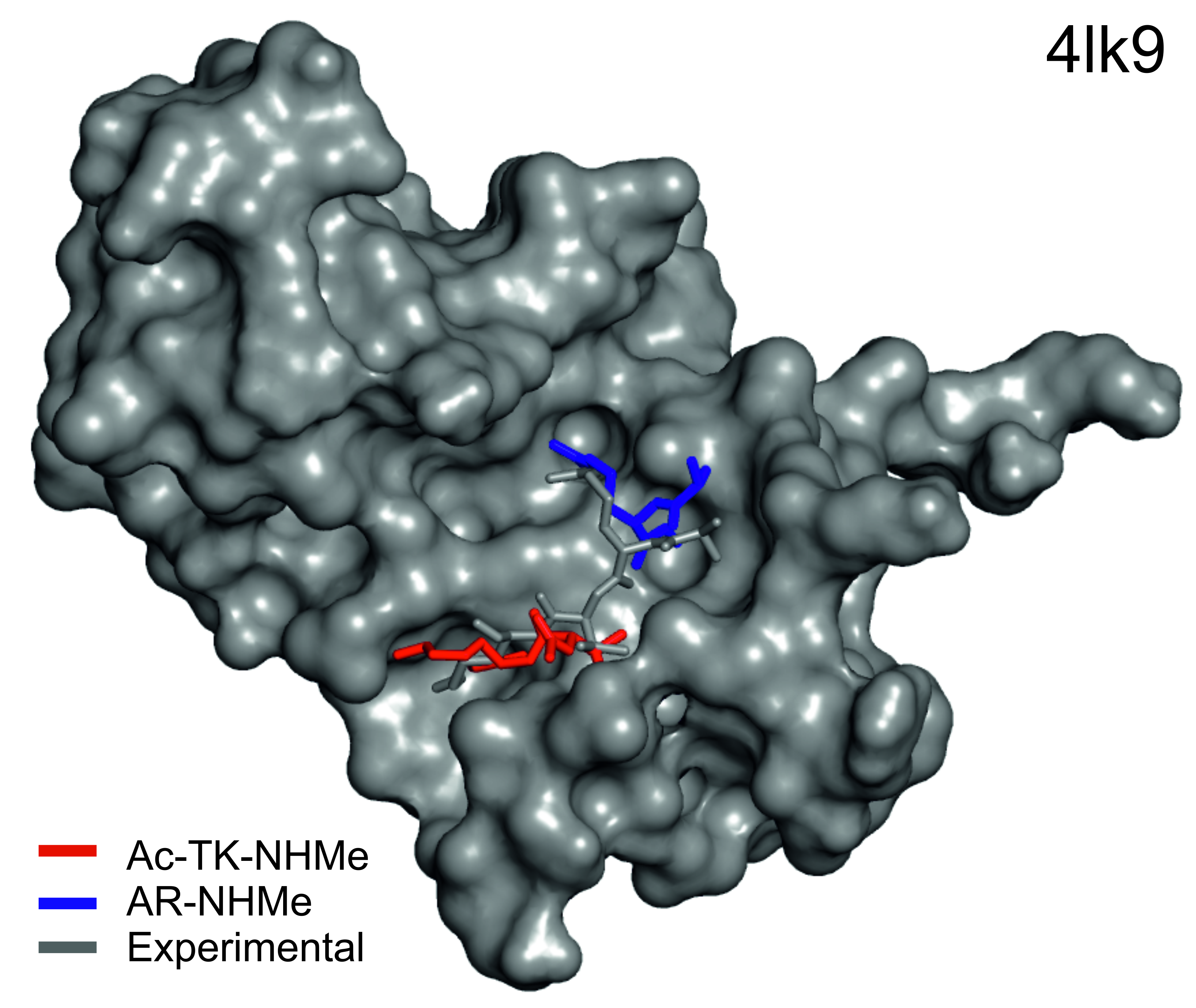

Supplement: Supplementary file 1 [file ijms-20-00422-s001.zip › ijms-420522 suppl revised/supplementary_figs/supplementary_fig_4.tif]

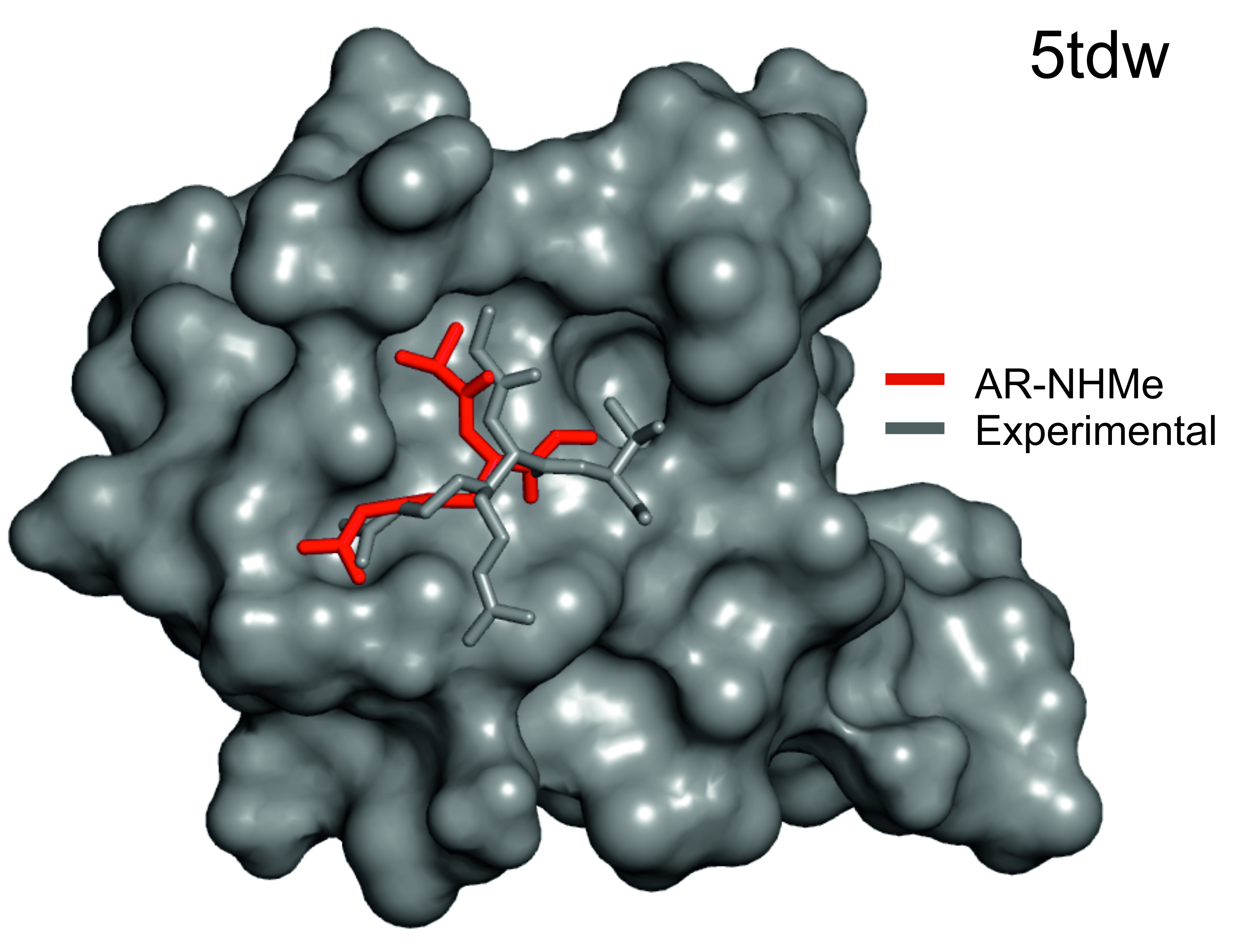

Supplement: Supplementary file 1 [file ijms-20-00422-s001.zip › ijms-420522 suppl revised/supplementary_figs/supplementary_fig_5.tif]
